# Supplementary material for: Association between physical activity and health-related quality of life in middle-aged and elderly individuals with musculoskeletal disorders: Findings from a national cross-sectional study in Korea
Source: PLoS One. 2023 Nov 16;18(11):e0294602. doi: 10.1371/journal.pone.0294602 (PMC10653435; doi:10.1371/journal.pone.0294602)
Supplement: Attachment — Submitted filename: Point-by-point response.docx [file pone.0294602.s001.docx]

**Revision Note for Manuscript ID: PONE-D-23-15070**

**Association between physical activity and health-related quality of life in middle-aged and elderly individuals with musculoskeletal disorders: Findings from a national cross-sectional study in Korea**

We thank you for giving us the opportunity to revise our paper. In revising our paper, we have carefully considered the comments and suggestions of editor and reviewers, and have done our best to incorporate them accordingly. As instructed, we have attempted to explain the changes made in reaction to all of the reviewer’s comments. The reviewer’s comments were very helpful overall, and we appreciate the constructive feedback on our submission. After addressing the issues raised, we feel the quality of the paper has greatly improved and we hope you agree. Our response to each point is as follows, and we attach a revised manuscript with the track changes. Again, thank you for the valuable and helpful comments.

**Response to Reviewer #1’s comments**

**Point 1**

There is mistake in total number of participants in the Abstract and Data and study population section.

- *We are very sorry for the confusion. There was a typo, and the number of study participants was all corrected as follows. Thank you for your careful review.*

**Revised manuscript with track changes, page 2 & 5:** 4,731 participants (783 men and 3,948 women)

**Point 2**

The conclusions in Abstract are not supported by study results. Based on cross-sectional nature of research it is not possible to conclude that physical activity can improve HRQoL. It should be corrected in the whole manuscript.

- *It seems that our conclusions have been somewhat exaggerated. Based on your valuable comments, conclusions have been rewritten based on the results of the study. This has been corrected throughout the paper including the abstract.*

**Revised manuscript with track changes, page 2:** Our findings suggest that engaging in regular physical activity contributes to preventing exacerbation of HRQoL, even if the individual suffers from musculoskeletal disorders.

**Revised manuscript with track changes, page 12:** 2) Leisure-related moderate-intensity physical activity had the greatest effect on preventing exacerbation of HRQoL.

**Revised manuscript with track changes, page 14:** In conclusion, our findings identified that engaging in regular physical activity contributes to preventing exacerbation of HRQoL, even if the individual suffers from musculoskeletal disorders.

**Point 3**

In Measures section, lines 110 – 111 should be placed in Results section.

- *Based on your meaningful comment, information on the range of the EQ-5D index and the average value of men and women has been replaced to the results section.*

**Revised manuscript with track changes, page 8:** The average EQ-5D index for the participants in this study was 0.861 for men and 0.849 for women. Therefore, 66.5% of men and 65.6 of women had EQ-5D values above average and good HRQoL.

**Point 4**

In lines 111-114 is explained the procedure of categorizing participants into groups with poor HRQoL and good HRQoL. It is unclear why participants with “below-average EQ-5D index” are categorized as “poor HRQoL” if “A response of “1” for all five dimensions indicates the best state of health…”. Explanation of scoring protocol indicate that lower result is better result. Please explain and/or correct procedure of categorizing participants into 2 groups according to their HRQoL.

- *Thank you for pointing out the important part, and we apologize for providing insufficient information about EQ-5D index calculation and HRQoL definition. The procedure of categorizing study participants into 2 groups according to HRQoL was again accurately explained.*

**Revised manuscript with track changes, page 6:** A response of “1” for all five dimensions indicates the best state of health, and the EQ-D value in this case is defined as 1. On the other hand, if there is a response of “2” or “3”, EQ-5D is calculated as 1 – quality of weight. The worst state of health is indicated when the responses of all five dimensions are “3”, and the EQ-5D value in this case is -0.171. Therefore, the EQ-5D index has a value of -0.0171 to 1. That is, the smaller the EQ-5D value, the worse the health status.

**Point 5**

Line 115: “The variable of interest was physical exercise…” This is not correct because GPAQ was used to assess total physical activity not exercise. Please correct.

- *Thanks for your valuable comment. We have corrected all of these expressions in the manuscript.*

**Point 6**

In Measures section it is not explained how participants were categorized into 3 groups according to their physical activity level. It is only explained cut-off point for “regular physical activity”. Please add explanation of cut-off points for other two groups.

- *Thanks for your meaningful comments. We have detalied the criteria for categorizeing participants into 3 groups according to the lecel of physical activity as follows.*

**Revised manuscript with track changes, page 7:** Accordingly, in our study, those who met the above criteria were defined as the regular physical activity group. On the other hand, those who could not calculate METs by engaging in physical activity for less than 10 minutes in the last week were classified as the no physical activity group, and those who did physical activity but not meet the above criteria were classified as the irregular physical activity group.

**Point 7**

Line 147: number of participants should be corrected in the whole text. The number of participants is differently presented in the Abstract and Data and study population section.

- *We are very sorry for the confusion. There was a typo, and the number of study participants was all corrected as follows. Thank you for your careful review.*

**Revised manuscript with track changes, page 8:** Among the total study sample(N=4,731), 783 (16.6%) were men, and 3,948 (86.4%) were women.

**Point 8**

In Table 1 it is written “Bad EQ-5D score” although in Methods section of the manuscript is was called “poor EQ-5D score”. Please correct.

- *We are very sorry for the confusion. Based on your comment, we have corrected the expression.*

**Point 9**

In Table 2 all variables, except for physical activity, are irrelevant for the presented study. I think it should be excluded from the Table 2.

- *Thanks for your valuable comment. We agree with you and have rewritten Table2 as follows.*

| **Revised manuscript with track changes, page 10:**  **Table2. Results of factors associated with Health-related quality of life (HRQoL)** | | | | | | | | | | |
| --- | --- | --- | --- | --- | --- | --- | --- | --- | --- | --- |
| **Variables** | | **Men** | | | |  | **Women** | | | |
|  |  | **EQ-5D score < Mean**^*^ | | | |  | **EQ-5D score < Mean**^*^ | | | |
|  |  | **OR** | **95% CI** | | |  | **OR** | **95% CI** | | |
| **Physical activity** | |  |  |  |  |  |  |  |  |  |
|  | None | 1.00 |  |  |  |  | 1.00 |  |  |  |
|  | Irregular practice group | 0.99 | (0.58 | - | 1.68) |  | 0.77 | (0.62 | - | 0.96) |
|  | Regular practice group | 0.58 | (0.37 | - | 0.90) |  | 0.64 | (0.53 | - | 0.79) |
| ^* The mean of the EQ-5D index (Men: 0.861; Women: 0.849)^ | | |  |  |  |  |  |  |  |  |

**Point 10**

In Table 3 is presented subgroup analysis stratified by the type and intensity of physical activity but it is not clear how participants were categorized in subgroups of physical activity. What were the cut-off points? How were the groups defined? It does not make sense to compare groups if some of participants can belong to more than one group. Please explain how you categorized participants and explain the meaning of this results.

- *Thanks for your meaningful insight. We will explain how we defined each group. First of all, the type of physical activity is the self-reported cotent of the physical activity that participnatns mainly engage in their daily life. In addition, they were asked to respond to the intensity of the physical acitivity they engaged in, and the definition of intensity was defined as follow, already mentioned in the measures section.*

*“High-intensity activity is vigorous physical activity that results in breathlessness or an increased heart rate; it is rated at 8.0 METs. Moderate-intensity activity refers to moderate physical activity that results in slight shortness of breath or slightly increased heart rate; it is rated at 4.0 METs.”*

*The definitions of moderate and high-intenstiy physical activity were explained to the participants in advance and they responded. Finally, three types of physical activity and two levels of intensity were combined and categorized, and this was an analysis conducted to explore wich type of activity and at which intensity would be most effective in preventing HRQoL deterioration. The group that does not exercise at all (None) is determined as the reference group, and the activity of each combination is compared to with None group. We hope this answer has been sufficient.*

**Point 11**

Additional table 1 and 2 are missing from the manuscript.

- *We are very sorry for missing additional tables. We will reattach these as file.*

**Point 12**

In Discussion section results are put in the context of very few previous studies neither without explanation of possible reasons for determined association nor possible implications of findings in terms of public health interventions and recommendations for physical activity programs tailored to the identified population.

- *Thanks for the meaningful insight. We aggree with you and have revised the Discussion section throughout. First, we added a review of previous studies that can support our findings. In addtion, after further explaining the limitations of our study, the conclusions are also rewritten.*

**Revised manuscript with track changes, page 12:** Our findings are consistent with the existing evidence that lack of physical activity and activity limitation are fetal physical and psychological risk factors for the management of osteoarthritis and are associated with HRQoL. Several previous studies demonstrated that interventions related to exercise and weight control are effective in controlling symptoms and maintaining quality of life in patients with osteoarthritis.

**Revised manuscript with track changes, page 12:** Their findings, which demonstrated that physical activity positively affects HRQoL in patients with musculoskeletal disorders, are consistent with our findings. Although they assessed only one type or intensity of exercise, we identified the most effective combination of exercise type and intensity to enhance patients' HRQoL and suggested practical applications.

**Revised manuscript with track changes, page 13:** Third, as our study focused on individuals diagnosed with musculoskeletal disorders, patients who were unable to engage in regular exercise due to advanced musculoskeletal disorders were also included in the analysis. Therefore, the size of association may have been underestimated. Due to the nature of the cross-sectional study, inverse associations are also possible, so the results should be interpreted cautiously.

**Revised manuscript with track changes, page 14:** In conclusion, our findings identified that engaging in regular physical activity contributes to prevention exacerbation of HRQoL, even if the individual suffers from musculoskeletal disorders. Especially noteworthy was the significant association found between leisure-related moderate-intensity activity and high HRQoL, suggesting that a leisurely exercise program that musculoskeletal patients can enjoy should be provided to improve their health-related quality of life. It should be provided manuals of the appropriate type and intensity in consideration of the patients’ pain and severity.
