# Supplementary material for: Association between physical activity and health-related quality of life in middle-aged and elderly individuals with musculoskeletal disorders: Findings from a national cross-sectional study in Korea
Source: PLoS One. 2023 Nov 16;18(11):e0294602. doi: 10.1371/journal.pone.0294602 (PMC10653435; doi:10.1371/journal.pone.0294602)
Supplement: Attachment — Submitted filename: Point-by-point response (round2).docx [file pone.0294602.s002.docx]

**Revision Note for Manuscript ID: PONE-D-23-15070R1**

**Association between physical activity and health-related quality of life in middle-aged and elderly individuals with musculoskeletal disorders: Findings from a national cross-sectional study in Korea**

**Response to Reviewer’s comments**

The authors have responded to the previous reviewer's comments and used these comments to revised their work. However, there are still some problems that need to be fixed.

- *Thank you for the valuable and helpful comments. Our response to each point is as follows, and we attach a revised manuscript with the track changes and clean version file.*

**Point 1:** It seems that the authors uploaded the original version as the clean version for the resubmission. Specifically, the numbers are still wrong in the Abstract for the clean version, although the tracked change version has corrected the typos. The authors should recheck again regarding their data accuracy and the uploaded materials.

- *Based on your comments, we’ve rechecked the uploaded materials again. We attached a revised manuscript with track changes and clean version file.*

**Point 2:** The authors still have some unclear information regarding the data. For example, The authors said, "The total survey population from the recent five years (2016–2020) included 39,738 individuals. Among these individuals, we only included middle-aged and elderly individuals over 40 years of age who were diagnosed with one or more of the following conditions in the analysis: osteoarthritis, rheumatism, and osteoporosis. After excluding missing data (N=41), 4,731 participants (783 men; 3,948 women) constituted the study sample." It is unclear how many participants were available for those age over 40 years. From 39,738 to 4,731 is large reduction. Therefore, it has to be clear to indicate the data cleaning process. It will be even better if the authors could have a flow chart to indicate how they remove the participants from the 39,738 to the final 4,731.

- *We apologize for not clearly explaining the sample selection process. According to your valuable comment, we have revised the main text to accurately state the number of participants excluded from the analysis of this study.*

**Revised manuscript (clean version), page 5, line 99-102:** Therefore, those under 40 years of age (*N*=16,483) and those without musculoskeletal diseases (*N*=18,483) were excluded. Finally, after excluding missing data (*N*=41), 4,731 participants (783 men; 3,948 women) constituted the study sample.

**Point 3:** Following the previous comment, the present sample includes a large proportion of female participants. Is this the case for Korean's gender distribution for middle-aged and elderly individuals with musculoskeletal disorders?

- *Thank you for asking such an important question. As a result of investigated the general characteristic of patients with musculoskeletal disorders in Korea, it was found that there were about 1.5 times more female patients than male patients. Moreover, looking at the distribution by age, patients in their 50s or older accounted for about half of all patients.*

**Point 4 :** When mentioning the interventions on physical activity improvement, please also indicate the potential programs on education and smartphone use. Please see the following papers.

- *Based on your valuable comment, we have supplemented the policy implications of this study by referring to the recommended references.*

**Revised manuscript (clean version), page 14, line 245-248:** Furthermore, interventions to improve physical activity need to be introduced based on smartphones and wearable devices to increase accessibility, and education on manuals is needed to increase participation in interventions.
